# Psychiatry's need for Vergangenheitsbewältigung: ‘culture wars’, cognitive dissonance and coming to terms with the past

**DOI:** 10.1192/bjo.2022.607

**Published:** 2022-11-22

**Authors:** Peter Lepping, Rob Poole

**Affiliations:** Betsi Cadwaladr University Health Board, Wrexham, Wales; Centre for Mental Health and Society, Bangor University, Wales; and Mysore Medical College and Research Institute, Mysuru, India; Bangor University, Wales

**Keywords:** Vergangenheitsbewӓltigung, psychiatry's past, history of Psychiatry, coming to terms, society

## Abstract

UK psychiatry's sense of self rests on being part of a socially progressive national tradition. This makes it difficult to engage with more critical narratives. The process of analysing and accepting psychiatry's past can help our profession to get closer to its real self and on a path to a better future.

In recent years, UK psychiatry has made tentative steps to acknowledge some of the shameful episodes from its past, from aversion therapy for homosexuality to institutional racism. Frank discussion of these aspects of our profession's history causes a degree of cognitive dissonance or even offence to some colleagues, and we fully acknowledge this. It is hard to avoid defensiveness in response to shaming facts, even when they are perceived to belong in the remote past. This is one of the reasons psychiatry has difficulty in responding constructively to its critics. Here, we outline why we believe that it is important to have this discussion, despite mixed responses within the profession. We explore some ways to approach our collective cognitive dissonance in order to learn from our past and to truly come to terms with it, individually, as a profession, and as a society. We single out psychiatry because we are psychiatrists. Other medical specialties and scientific disciplines face their own struggles to acknowledge the impact of shameful past episodes on the present.

## Acknowledging the past

Organised psychiatry in the UK has started to face up to its past, both distant and recent. The Royal College of Psychiatrists has taken steps to acknowledge racism in the clinical practice of psychiatry, to challenge the imperial legacy in the psychiatric curriculum, and to address the discrimination experienced by Black and minority ethnic psychiatrists. Similarly, in 2017, the then President of the Royal College of Psychiatrists, Dr Wendy Burn, issued an unequivocal apology for psychiatrists’ involvement in the use of aversion techniques to ‘cure’ gay sexual orientation. These are welcome developments, but they are just the start. Meaningful change necessitates a thorough and continuing process to eliminate the continuing effects of past abuses. We need to fully acknowledge them, including the effects on those abused. This demands tangible action in the present.

German psychiatry has made a belated but concerted effort to come to terms with the profession's involvement in the crimes of the Nazi regime.^1^ The UK was a victor in the Second World War, and British psychiatry has long avoided examination of its direct involvement in the development of eugenics that was later used to justify the mistreatment, sterilisation or murder of large numbers of people with learning disabilities and chronic mental illness across Europe and North America.

## Psychiatry, empire, eugenics and hereditary degeneration

The term ‘eugenics’ was coined by Sir Francis Galton, and the Eugenics Laboratory he founded at University College London was the key institution in the world for the development of concepts of genetic (or racial) hygiene. He classified the ‘comparative worth of different races’ into grades from A to I, arguing that Africans could never attain the average grade of Anglo-Saxons.^2^ In 2021, after a lengthy investigation, University College London formally acknowledged this and made an apology, de-naming spaces on its campus that previously honoured Galton and another main advocate of eugenics, Karl Pearson (who devised many of the biometric and statistical techniques still in use today, and who propagated the idea of ‘social imperialism’). British psychiatry and psychology had close involvement in the eugenics project. Galton, for instance, worked in close collaboration with psychologists and psychiatrists at Royal Bethlem Hospital, including efforts to use photographs of patients to delineate physical signs of hereditary degeneration.^3^ Galton's ardent admirer Karl Pearson noted, without criticism, how Galton's views on genetics and eugenics shaped his views of patients and society. Pearson wrote in 1930 in a book about Galton: ‘And we ask why, with a common environment, does one man achieve and another fail to do so? The answer can only be: Such is the law of inheritance, and that was Galton's answer’.^4^

Henry Maudsley, who bequeathed the hospital named after him, was the key UK proponent of the concept that mental illness was an untreatable genetic disorder leading to ‘hereditary degeneration’ across generations.^5^ In addition, he used anatomical studies to claim that ‘the brain of the Negro […] does not reach the level of the white man's brain’.^6^ He continued to point out that ‘even’ European females had heavier brains [than Black Africans and Aborigines] and proceeded to find alleged similarities between the brains of Africans and orang-utans.^7^ In 2022, Hilton and Freudenthal argued that Maudsley deliberately misrepresented Friedrich Tiedemann,^7^ an anatomy professor from Heidelberg, Germany, whose research had suggested in 1837 that ‘neither anatomy nor physiology can justify our placing of [Black people] beneath the Europeans morally or intellectually’.^8^ Maudsley called Tiedemann the ‘Negro's advocate’ in this context.^6,7^ Although modern psychiatry ought not to consider comparisons of anatomy or physiology to be relevant to the human value or abilities of different peoples, it shows that even at the time when they *were* considered relevant, there were differing views, and Maudsley was unequivocally in the camp of those who used the eugenic ideas of the time to justify assertions of White superiority. In the 19th century, ideas of hereditary degeneration and eugenics spread quickly in the wider population. They were intimately linked to contemporaneous ideas about racial hygiene, and they resulted in the therapeutic nihilism that tainted mental hospitals for decades, encouraging excesses such as insulin coma and deep narcosis treatments.

Eugenics developed contemporaneously with the ‘scramble for Africa’, which was the most rapid period of British imperial annexation of territory and subjugation of foreign populations. The eugenic ideology of racial superiority was used to justify imperial expansion,^9^ with explicit comparisons made between inferior peoples and hereditary degeneration among the lower classes, who were disproportionately represented in mental hospital populations. In our opinion, the legacy of colonial racism is seen in the modern-day disproportionate use of coercion in mental health services interactions with patients of African heritage.^10^ British psychology was equally heavily engaged in the eugenics project. Modern critics such as David Marks argue that ‘from the perspective of the colonial British Empire, the eugenics mission was an emblem of ‘white supremacy’, the traces of which seep into the British Psychological Society to the present day’.^11^ Marks states that institutional racism and micro-aggression against non-White psychologists are still active in the British Psychological Society, citing several recent controversies, and that racial stereotypes from the colonial past have a residual impact on psychologists’ practice and research. As psychiatrists, we wish to focus on our own profession, but Marks's article underlines that similar imperial themes affect other scientific disciplines.

Psychiatrists often react defensively to the suggestion that mental health services are structurally racist, even though this does not imply that psychiatrists are necessarily individually racist. However, in our opinion, there is an obvious link between imperialism, White supremacism, concepts of eugenics, hereditary degeneration and modern structural racism. Even psychiatrists who do not agree that psychiatry bears some culpability for eugenics must acknowledge that late Victorian psychiatrists could not have remained unaffected by the pervasive doctrines of British racial superiority of their time, and that modern psychiatry was built on these unsound foundations. This defensiveness may extend to psychiatrists from non-White backgrounds. The reasons for this are complex, and they cannot be appropriately explored by two male White psychiatrists in a brief article.

## Neo-liberalism and culture wars

In the last few decades, we have increasingly seen movements across the world that challenge dominant historical narratives. This is particularly uncomfortable for the UK because of our own colonial and imperial past. British politicians and media continue to project a positive image of the British Empire, construing it as the time when Britain was ‘Great’. The British supposedly civilised the world through propagation of their civil institutions and legal system. Injunctions to be proud of the Empire are commonly accompanied by denial of the oppressive and destructive consequences of domination of one country by another. Surveys suggest that only the Dutch surpass the British in their insightless pride in their imperial past.^12^

The Second World War and the Empire arguably form the strongest elements in the nationalist narrative of White Britain, reinforced in the education system, political rhetoric, the media and entertainment. Voices that are critical of this benign and victorious historical narrative provoke furious condemnation from authority and the media.^13^ The British-Nigerian journalist David Olusoga puts it thus: ‘If you have been told a version of your history and that is part of your identity, it's very difficult when people like me come along and say: ‘There are these chapters [that you need to know about].’ People feel – wrongly in my view – that their history is being undermined by my history. But my history isn't a threat to your history. My history is part of your history’.^13^ From the point of view of the peoples that have been subjugated, all empires are built on racism, oppression, misappropriation and exploitation. Despite strong support from the historical record, pointing this out provokes defensive counter-attack and a refusal to engage with alternative views. This is seen in negative reactions to the Black Lives Matter movement. Such diverging voices therefore have difficulty in finding a platform in popular media. This prevailing narrative has not only had a profound influence on recent political decisions, but it has also provoked a backlash by British people from ethnic minorities and many young people in general, who feel that their values are not represented by this complacent conservative stance.

The binding of British patriotism to pride in the Empire has become a battle cry for those who wage so-called ‘culture wars’, where voices that dissent from the narrative accepted by the political right are defined as non-patriotic and thus anti-British. Interestingly, dissent from the prevailing narrative has always been acknowledged, negating the idea that values were fundamentally different in the 19th and early 20th centuries, and that any judgement of past actions is therefore ahistorical.

A good example of this is the aftermath of the Amritsar (Jallianwala Bagh) massacre, which took place on the 13 April 1919. At the time, many people in Britain were highly critical of Brigadier General Dyer's decision to shoot hundreds of unarmed and peaceful civilians in cold blood. However, apologists and supporters of Dyer immediately started a successful public rehabilitation campaign,^14^ the tone of which was remarkably similar to today's so-called ‘culture wars’. We fully acknowledge that historical events can be seen in different ways, some that focus on events and some that use a lens of values, either past or present. Criticism of the actions of Empire or past psychiatrists are not new or in any way restricted to modern interpretations of human rights. The instinctive intellectual response is to excuse past wrongdoing on the basis that values were different then. In reality, there was a range of opinion and values at the time, and our perceptions of the values of the past have been strongly influenced by those who control historical accounts, among them many apologists of Empire. Real harms occurred, even if perpetrators were insensitive to them at the time. The many dissenting contemporaneous voices negate the idea that people in the past adhered to a single monolithic ethic that supported the dominant view.

## The past is alive in the present

In sharp contrast to present political rhetoric in Britain, the reputation of the British Empire in its former colonies is nothing like as charitable.^14^ Furthermore, the consequences of Empire for British society today are significant. As the British journalist and author Sathnam Sanghera puts it: ‘The manner in which our imperial history inspires a sense of exceptionalism results in dysfunctional politics and disastrous decision-making. Our collective amnesia about the fact that we were, as a nation, wilfully white supremacist and occasionally genocidal, and our failure to understand how this informs modern-day racism, are catastrophic’.^9^ It is because the imperial narrative still matters today that psychiatry ought to take note. Victorian attitudes towards race were shaped by a eugenic science that was flawed and ideologically distorted, to which psychiatry significantly contributed. Being part of the narrative makes it more difficult for psychiatrists to fully acknowledge its consequences in the here and now. In keeping with this, UK psychiatry has invested much of its pride and sense of self in being part of a national tradition that considers itself to be socially progressive. The Royal College of Psychiatrists’ recent pamphlet *Celebrating Our History* demonstrates this, focusing on the organisation's progress towards inclusivity while attributing deviations to the failings of individuals. An unwillingness to recognise systematic failings makes it more difficult to engage readily with other narratives or to acknowledge challenging facts. A deeper analysis creates cognitive dissonance over who we are and over our role within the life of the nation. This is hard to bear, and it is more comfortable to simply reject any re-evaluation of the foundations of our professional pride.

## Vergangenheitsbewältigung

‘Coming to terms with one's past’ may be as good a translation as we can make for the German word *Vergangenheitsbewältigung*. It describes the process of analysing and accepting one's past with all its failures, flaws and moral ambiguities. The aim is to get closer to one's real self, learn from past mistakes and develop concepts for a better future. The concept is valuable for individuals and professions as well as for countries or nations. Germany went through this process after the Second World War. Many of these stories are full of personal guilt and responsibility, moral injury, shame and suffering. They are about collective failure as well as collectively building a better future.

The initial dilemma is the tension between the self-esteem we get from being part of a benign national and professional narrative and the cognitive dissonance that will be caused and has to be worked through when we challenge this narrative. While we fully recognise and personally experience this dissonance, if we are serious in our remorse for past wrongdoings, we must find a way to engage with historical truths that allows us to come to terms with our own, our country's and our profession's pasts, while preserving self-esteem. In a psychotherapeutic sense, shame needs to be integrated and not sequestrated. The philosopher Cioran believed that failures are much more important than successes because, when analysed, they get much closer to one's actual true self, allowing a person or a nation to come to terms with the past and learn from it for the future.^15^ This can be painful, which makes it important to see the process itself as something positive, leading to a more authentic professional self-awareness.

A *Vergangenheitsbewältigung* process of coming to terms with the past might become a source of a new sense of self, integrity and pride. An honest analysis that acknowledges past failures can build a better future. There are a number of possible ways in which this can happen. Sathnam Sanghera and others argue for a broadening of the national curricula in schools and universities^9^ to widen our understanding of our own past with regard to Empire. This should not only include the suffering in the colonies at the hand of the British and their colonial allies but also the negative consequences of Empire in the UK, given that the wealth the Empire created was only shared among a few, usually already wealthy, people. Psychiatry may equally need to widen its curricula to acknowledge the impact up to today of its involvement in eugenics, homophobia and dangerous, ineffective treatments such as insulin coma therapy. Telling the complete story can be part of a process that helps us come to terms with the shameful aspects of our past without losing self-esteem. Accepting our complex past in its entirety and learning from it can be a source of pride.

Psychiatry has special understanding of self-esteem, shame, stigma and the development of the self. Furthermore, whether we like it or not, psychiatrists exercise authority on behalf of the state. If we can examine and come to terms with shameful aspects of our past and present, we may have something to say to the nation about how to deal with past failings. This includes a willingness to learn about and acknowledge what has happened and to act on that knowledge to make things different in the future. Just like coming to terms with personal histories, analysing professional and national failures, and not just successes, can show a route to a more mature society. It can help heal the power difference in doctor–patient relationships and lead to an improvement in the relationship between psychiatrists and those who use our services. A critical part of this process is to find space to examine this as individuals and as a profession, in order to come to terms with our failures and move forward.
